# Love Island and Relationship Education

**DOI:** 10.3389/fsoc.2019.00079

**Published:** 2020-01-23

**Authors:** Janette Porter, Kay Standing

**Affiliations:** School of Humanities and Social Science, Liverpool John Moores University, Liverpool, United Kingdom

**Keywords:** reality TV, domestic abuse, young people, gaslighting, relationship education

## Abstract

The rise of reality TV programmes focussing on relationships and the search for “love” has focussed media attention on the portrayal of healthy relationships, gender roles, and intimate partner abuse (IPVA). Love Island, a UK reality TV programme, was watched in 2019 by over 3 million viewers, a majority of whom are young women aged 16–34, though a younger teenage demographic also watch. Many of these younger viewers may be learning about what healthy relationships are like, and entering their first romantic relationships. Contestant's behavior on Love Island prompted Women's Aid to issue a statement speaking out against unhealthy behaviors in relationships—especially “gaslighting,” a form of emotional abuse that makes someone question their own feelings, memories, and version of reality. Based on our experience of running a relationship education program in 24 schools, as part of the Tender national partnership and our research with young people on their perceptions of Love Island, the paper will examine the role reality TV programmes play in young people's understandings of healthy relationships. It argues representations of relationships on Love Island are framed within normative heterosexuality, and enables the normalization of emotional abuse. However, we also argue that these programmes can be a catalyst for discussion amongst young people and open up spaces, especially online, to challenge dominant constructions of relationships. It also makes recommendations for education policy and practice around relationship and sex education in schools.

## Introduction

The 2019 series of Love Island^1^, a UK reality TV dating show, had an average audience of 3.63 million, peaking at 4.05 million for the show's finale, and gained a 21.4% average audience share (BBC News, 2019a). The viewing is both gendered and generational. The 2018 series 3 of Love Island attracted more than half (52.3%) of all television viewing by the 16–24 age group (Jones, 2017), most of which were young women aged 16–34. However, a younger audience also watches, with evidence of primary school children (aged 8–11) viewing the post watershed programme (BBC News, 2019b). Whilst this has raised concerns about the adult content of the programme, it also raises the possibility of the use of programmes such as Love Island to start age appropriate conversations about relationships with younger viewers. Many of these younger viewers may be entering their first romantic and sexual relationships, and the models of relationships shown on the programme can be problematic. The behavior of contestants on the last two series of Love Island, 2018 and 2019, prompted Women's Aid, a UK domestic abuse charity, to issue statements speaking out against the unhealthy behaviors in relationships shown on the programme, in particular “gaslighting,” a form of emotional abuse that makes someone question their own feelings, memories, and version of reality.

For young people it is argued that this behavior may influence their understandings of healthy and unhealthy relationships as it normalizes abuse in relationships. This is an important issue as research suggests that one in four teenagers state they are more influenced by celebrities than other people they know (Giles and Maltby, 2004), and national and international evidence demonstrates that abuse and violence in young people's relationships represents a substantial problem (Barter et al., 2009; Fox et al., 2014; Stonard et al., 2014; Young et al., 2017). Research from Europe (STIR, 2015b; Young et al., 2017) demonstrates that adolescents and young people are at particular risk of intimate partner violence and abuse (IVPA) and evidence indicates that victimization is typically higher among young women than young men (Barter et al., 2009, 2015). Emotional partner abuse is a common experience among young people; research shows that nearly three quarters of teenage girls, and half of teenage boys report some form of emotional partner abuse in relationships (STIR, 2015b). However, Barter et al. (2009) argues that teenagers do not recognize the unhealthy behavior as abuse and therefore do not report it to anyone.

The UK government has recognized the role of TV, celebrities, and social media in promoting healthy relationships and challenging IVPA. These include Disrespect Nobody^2^ and This is Abuse campaigns which partnered with the Channel 4 teen soap Hollyoaks in 2013, to target 13–18-year-olds with extensive media coverage.

There is therefore a need for positive role models of healthy relationships in the media and within the school curriculum (Porter and Standing, 2018). The next section charts the rise of reality TV and its relationship with social media, enabling young people to interact and discuss in a way not previously possible for younger generations.

## The Rise of Dating Reality TV Shows and Social Media

Reality TV shows have become more popular since the late 1990s; the introduction of shows such as Big Brother in 2000^3^ with the rise of reality TV defining the millennial pop cultural landscape (Grazian, 2010). In 2016, reality shows were watched by 39% of adults in the UK, including 48% of women and 50% of people aged 25–34 (Barker et al., 2018).

Reality shows are cheap to produce and offer the “ordinary” person a chance to become “known” (Couldry, 2002; Holmes, 2004). The appeal of reality TV lies in the supposed “authenticity” of the participants, and the opportunities it offers, as Grindstaff and Murray (2015, p. 130) argue:

‘*Reality TV is largely effective as an industrial mode of persona production because it holds open the promise of moving an ordinary person from noncelebrity to celebrity status. Indeed, it is typically assumed that the main goal of getting on a reality program is to leverage ordinary participation into ordinary celebrity*.'

The audience sees the journey of self-transformation through which “ordinary” contestants find their “true self,” or their “true love” (Hill, 2002, 2004). These “authentic” experiences and true love is the plotline of shows such as The Bachelor and First Dates, which are popular amongst young female viewers. The concept of authenticity, of “being true to yourself,” is central to reality TV, and to the ultimate success of participants. The production of a particular individualized neoliberal identity or “selfhood” has been used by Ouellete and Hay (2008) to understand the fascination with authenticity in Reality TV, in which authenticity is viewed as a white Western construct of modernity (Feldman, 2014). Skeggs and Wood (2008) argue that reality TV promotes a neoliberal “subject of value” of middle-class selfhood, which makes moral judgements about behavior based on class, race, and gender. Celebrity discourses reflect and reproduce wider social attitudes whereby working class contestants are seen as “Other” (Tyler and Bennett, 2010) with “excessive and troublesome bodies and lifestyles” (Allen and Mendick, 2013, p. 463). These debates are particularly relevant when examining the gendered and heteronormative moral judgements around sexuality and healthy relationships.

Much of the literature on reality TV remains based in traditional TV viewing (Holmes and Jermyn, 2004). A new demographic of reality TV show viewers and participants are able to interact via social media in a way previously not seen, nor widely researched. Generation Z (Gen Z) is the generation of the internet, technology, and social media (Combi, 2015). The rise of celebrity culture (Turner, 2006, 2010) and the rise of social media influencers and Instagram culture (Okrent, 2017) mean participants subsequently become celebrities who create branded identities across multiple social media channels.

For Love Island and other Reality TV programmes, however, many participants are already “instafamous” social media influencers and are scouted by production teams, bringing further into question the “authenticity” and “ordinariness” of those taking part. For example, 30 out of the 36 2019 Love Island contestants were headhunted by ITV's casting team (Westbrook, 2019).

Marwick (2015, 2016) argues the notion of instafamous reinforces existing gendered hierarchies of fame, with “fit,” slim, white women having a higher rank in both traditional and social media than those who question this or do not fit the “ideal type.” Love Island presents a heteronormative image of the “perfect relationship” and an idealized white, slender, perfect-bodied individual as the “authentic” self. The colorism and misogynoir judgements made on reality TV have a long history, with black and BAME contestants consistently voted off early, and can be seen in the lack of diversity and early departure of the black female participants (Adegoke, 2018).

For audiences, reality TV has “coupled up” with social media to provide 24/7 content. In addition to the nightly programme, Love Island has an after show, an official love island podcast, YouTube channel, Instagram, a constant inflow of tweets and hashtags, the official love island app, chat rooms, forums, tumblers, and memes alongside other traditional media outlets. This enables the audience to interact with the programme in ways not previously seen with traditional media platforms. This possible effect and influence is an example of what Jones (2016) calls convergence culture (Jones, 2016 cited in Jenkins, 2016). What impact the flow of content across multiple media platforms, the cooperation of multiple media audiences, and the migratory behavior of media audiences has on young people's view on relationships is discussed below.

### Love Island, Social Media, and “Healthy” Relationships

The influence of reality TV on young people is contested, however, reality TV has historically been seen to incite moral panics (Thompson, 1998), and for Love Island, this is around sex and relationships, with media reports of the negative influence of Love Island on young people's self-esteem, body image, and sexual behaviors (Barr, 2019). Bilandzic (2006) argues that as people watch television, they slowly begin to absorb the ideas, views, and morals presented; she calls the television the best universal vehicle for passing on views and standards, and one that can also influence people to change their beliefs. Young people actively seek information on relationships and advice on the dating experience to help them navigate and guide their anticipations and beliefs (Ferris et al., 2007), and they list television, dating partners, parents, friends, and teachers/sex educators as their largest sources (Wood et al., 2002; Zurbriggen and Morgan, 2006). Past research suggests a link between viewing reality TV, and gender stereotyping, with watching reality TV associated with a strong adherence to traditional masculine ideology^4^ (Giaccardi et al., 2016), and a greater endorsement of gendered (hetero)sexual scripts for women, with sexual activity being seen as different for women and men, and in turn were associated with girls' lower sexual agency (Behm-Morawitz et al., 2015; Seabrook et al., 2017). van Oosten et al. (2017) found evidence that sexually oriented reality TV is one factor in young people's willingness to engage in casual sex (along with the internet, social media, and peers). However young people also state that these sources of information are not very accurate and have little effect on them, with parents and peers having the biggest impact on young people's attitudes to sex and relationships (Wood et al., 2002; Monahan et al., 2014). The influence of social media is also contested and contradictory; however, for Love Island's viewers, we argue it can be one mechanism through which they can discuss dating and relationships.

Love Island has been criticized for its portrayal and normalization of toxic masculinity^5^ (Petter, 2019), in particular emotional abuse in relationships, with gaslighting being a consistent problem. For example, Adam's^6^ manipulative behavior toward his partner Rosie in series three, which Rosie called out, prompted charity Women's Aid (2018) to issue a statement asking viewers to speak out against unhealthy behaviors in relationships (Porter and Standing, 2018). Several of the male contestants in series four repeated this behavior; for example Michael “dumped” his existing partner Amber to couple up with Joanna, and when confronted by Amber about his behavior, grew more aggressive in the face of her calm and reasoned questioning, calling her “childish” and “pathetic” and blamed her for problems in their relationship' (Verdier, 2019). Joe's controlling and possessive behavior toward Lucy, as he asked her not to spend time with fellow contestant Tommy, calling her behavior “disrespectful,” prompted another statement from Women's Aid, calling for the show's producers to be more aware of and end apparent “*unhealthy fledgling relationships [in the show] being used as entertainment*” (Women's Aid, 2019).

Toxic masculinity was also evident in several male contestants' behavior toward the female contestants seen as “difficult,” for example Michael's treatment of Amber discussed above. The sexual double standard was highlighted by the male contestants' reactions toward Maura's overt sexual confidence and agency^7^, which challenged normative views of female sexuality, which position women as passive, rather than sexual agents. Referring to her frequent discussion of sex in everyday conversations, before his date with Maura in the hideaway Tom commented ‘*it'll be interesting to see if she's all mouth or not”* which was overheard by Maura, causing her to confront Tom and call off the date. The other male contestants colluded in this as “banter” exposing the casual sexism in the villa.

Social media further highlighted the gendered double standards of “socially acceptable” sexual behavior, with Maura consistently portrayed in the media as sex obsessed and portraying “inappropriate behavior” for her comments such as: “I am having fanny flutters, I'm not even joking” (Newman, 2019), prompting headlines like the one below from The Mirror newspaper:


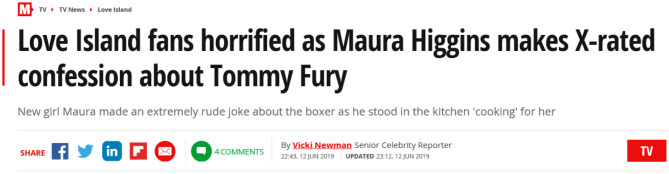


Her behavior toward both Tommy and Curtis raised issues around consent, as she kissed Tommy after he had had said no, and led to the hashtag #doublestandard.

There are other frequent examples of Love Island's women contestants being ‘slut shamed', of being labeled ‘sexually out of control' and being punished for this behavior (TanenBaum, 2015). In series two, contestant Zara Holland lost her Miss Great Britain crown after being shown having sex with Alex Bowen (no reprimand was given to Alex) and Megan Barton Hanson from series 3 continues to receive slut shaming comments on social media for her sexual choices (Barrett, 2019).

In contrast when Amy left the 2019 series voluntarily after her “half boyfriend” Curtis ended their relationship, saying “*I'll let you find whatever you were looking for*,” in order not to jeopardize his relationship with Maura, was seen as positive behavior, despite prioritizing men's emotional and sexual views at the expense of women's. This is an example of “himpathy” “the excessive or inappropriate sympathy extended to a male agent or wrongdoer over his female victim” (Manne, 2018; Buxton and Habgood-Coote, 2019), reinforcing gendered ideologies of female passivity.

Love Island does present some positive experiences, for example in showing positive female friendships; in 2019 the trending hashtag #friendship goals, with Maura advising Amy after her breakup with Curtis, and Anna confronting Michael over his behavior. It also show “bromances” and male friendships, for example in the 2017 series between contestants Chris Hughes and Kem Cetiney, and Jack Fincham and Alex George in 2018 (Wilkinson, 2019) which model healthy relationships.

Despite Love island presenting emotional abuse and hegemonic masculinity as normalized and as entertainment, there is evidence that audiences, including young people, reject the contestants who display those behaviors. The winner of series four, Amber, demonstrates both a rejection of toxic masculinity, and the importance of “authenticity” to audiences, with a recognition of her journey, as illustrated in the tweet below:


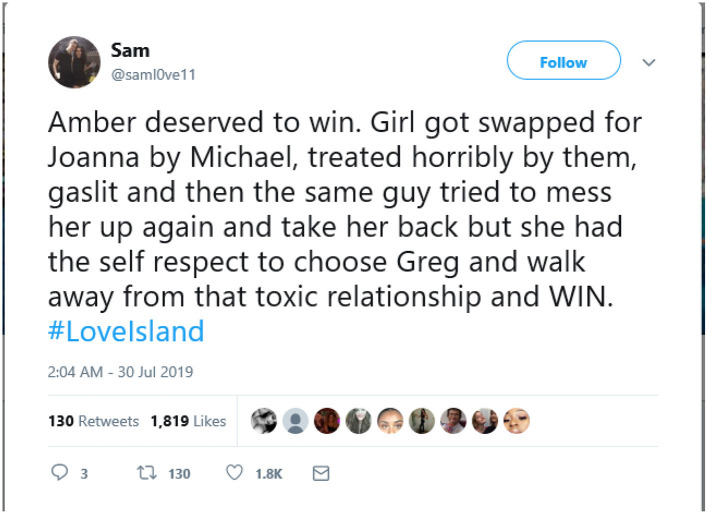


This also shows the influence of social media on audience's understandings of relationships, with young people recognizing, and rejecting, negative behaviors in contestants.

Whilst early reality TV programmes were broadcast for a limited period and thereby had a limited influence, newer audiences, such as Generation Z, have a constant flow of influences. The vast amount of fast-paced and interactive social media coverage means young people can engage with the shows, and that audiences can learn behaviors and absorb ideas from reality TV shows, thus underestimating or neglecting any debate on the programmes, which otherwise could have enabled them to challenge, instead of absorbing and copying, behaviors. If, as some commentators suggest, young people are getting some of their information about relationships from programmes such as Love Island, this can be a positive opportunity to discuss relationships both in and out of school.

The article goes on to discuss young people's views of relationships presented on Love Island to examine the role reality TV plays in their understanding of healthy and unhealthy relationships and if this can be used as a catalyst for discussion.

## Methods

This article draws on our experience of running relationship education workshops in schools with pupils aged between 13 and 16 years old and is supplemented with focus groups on Love Island with young people aged between 13 and 21.

Over a period of 6 years as part of the Tender National Partnership^8^, we worked in 24 schools across Greater Merseyside, UK, working with 3,158 pupils in total.

**Figure 1:**
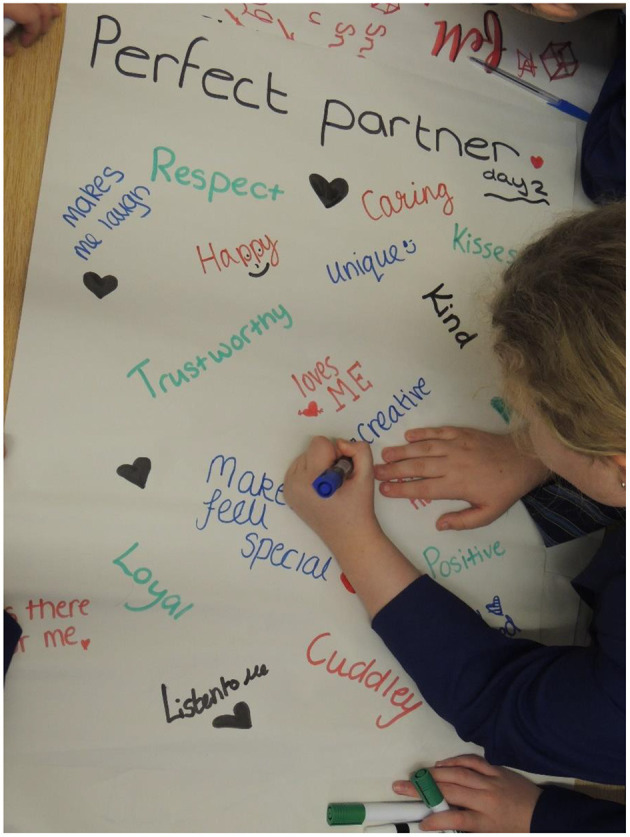
Young people explore ideals of their “perfect partner” informed consent was given for publication.

**Figure 2:**
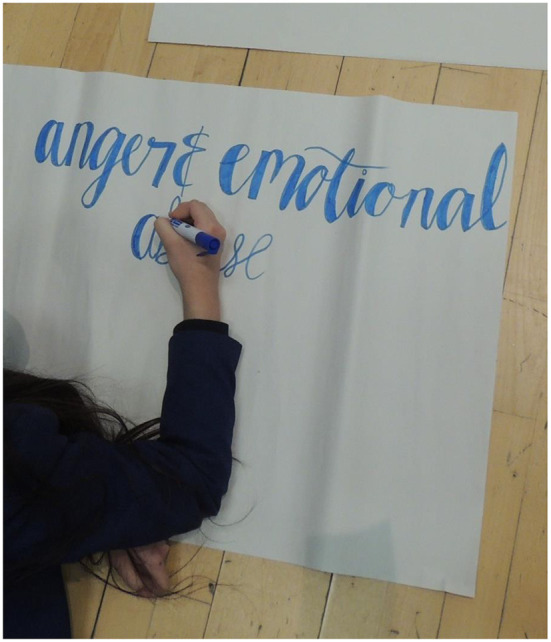
Exploring emotional abuse.

There is evidence that using creative approaches, such as arts or drama, as part of domestic abuse intervention/prevention projects is valued by, and has a positive impact on, students (Hester and Westmarland, 2005; Bell and Stanley, 2006; Pana and Lesta, 2012; Hester and Lilley, 2014; Sander-McDonagh et al., 2016). Stanley et al. (2015) in their evidence synthesis on prevention programmes in the UK, argue that drama-based interventions are highly valued by young people and experts, who argue that using dramatic approaches can create emotional intensity and contribute to what can be understood as “authenticity,” making interventions and key messages more “real” for young people. Figures 1, 2 show examples of young people on the project using creative arts based practice to explore “healthy” and “unhealthy” relationships as part of the Tender project.

**Figure 3:**
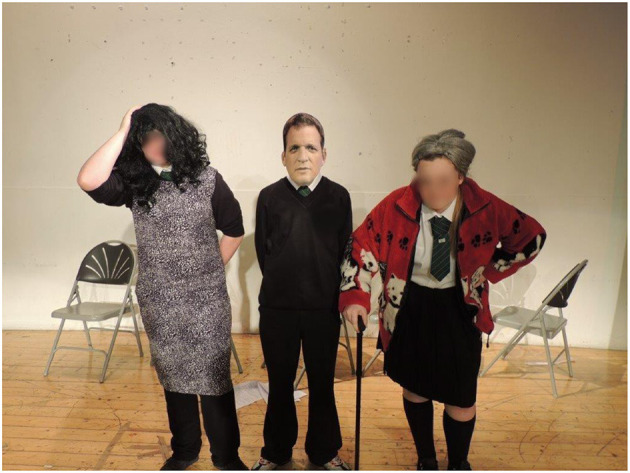
Young people acting out the Jeremy Kyle show as part of exploring relationship abuse.

From the beginning of the project reality TV was a reference and starting point for young people to discuss relationships. For example, in the early years of the project pupils in three of the schools suggested one way to resolve problems in relationships would be to go on reality TV shows such as the Jeremy Kyle show^9^, as Figure 3 shows. Young people acted out the Jeremy Kyle show to their peers to explore unhealthy relationships, saying ‘*we have a connection to it rather than just doing Grease or Hairspray' (School Group (SG) year 9 age 14 Female)* demonstrating the influence reality TV had on young people's understandings of relationships.

Focussed discussions and focus groups also played an important role in understanding young people's perceptions of healthy relationships. As part of the relationship education workshops we discussed Love Island with a group of year 9 (13–14 year olds) in a mixed school setting (*n* = 20) and year 8 female pupils (aged 12–13) in a single gender school (n-29). Sessions lasted approximately an hour, and notes were taken as part of the session. In addition to this, two focus groups in an out of school setting were conducted independent of the Tender project; one with young people aged 13–17 (*n* = 5), and one with young people aged 18–21 (*n* = 4).

Focus groups, along with the arts based methods used in the workshop, generate data through interaction (Kitzinger, 1994; Morgan, 1996, 1997) and are often used with young people to discuss sensitive topics such a sexual behavior and health (van Teijlingen et al., 2007). One of the drawbacks of focus groups is the potential influence of the facilitator, and as the focus groups, were a part of, or came after, the workshops on relationship education, participants may have more awareness of healthy and unhealthy relationships, thus influencing their answers.

Ethical approval was granted by the university; all participants were provided with written consent forms, as were their parents and guardians. Consent was obtained to publish participant verbatim quotes along with their ages and genders. We anonymized all the data, and referred only to young people's ages/school year and gender. Written notes were taken during focus groups and the facilitator's reflections from the workshops were recorded. We analyzed the data thematically (Braun and Clarke, 2006) to identify and analyse patterns linked to young people's understandings of healthy and unhealthy relationships. Three key themes emerged: the reasons why young people watch and the importance of social media, young people's understanding of the “fakeness” of the relationships shown on Love Island, and the potential for Love island to be used to start positive discussions about relationships. This paper now turns to explore these.

## Findings/Discussion

### Reasons to Watch: Social Media and Entertainment

Research shows some young viewers are influenced by what they see on reality TV shows (Zurbriggen and Morgan, 2006) and watching reality dating programs is positively correlated with holding gender stereotypical attitudes about dating and relationships, with some viewers using reality shows as informational guides for relationship behavior. Our research found that although one of the reasons young people watch reality TV is to seek advice and learn about relationships, it is not the biggest reason and, as Neimann-Lenz et al. (2018) argue, there are multiple reasons. The young people we spoke to stated their biggest reason was the entertainment value of the show, and the shared talking point with their friends:

“*[I] watch it because I have nothing else to do […. it's a conversation starter. And [for] entertainment purposes only and its comedic values” (Focus group (FG) age 17 Female.)*

Our research, although limited in terms of numbers, shows that young people have a more nuanced awareness of the debates around reality TV, and as Hill (2005) argues, viewers generally rejected the idea of learning from watching such shows and instead regarded them primarily as entertainment:

I honestly just watch it as entertainment and gossip” (FG age 16 F)

Many of the respondents noted the importance of social media on their viewing habits, suggesting the interconnectedness of different media platforms:

“We are watching it when we get to bed. You can know what's happening without watching it because everyone talks about it, as well as social media, Instagram, magazines, and newspapers.” (FG age 17 F)

Others did not watch the TV programme but because of the widespread social media coverage were still able to discuss it:

“*No [I don't watch it] but people talk to me about it….it's all over social media” (FG year 9 age 13 F)*

Social media is seen to make audiences active consumers, or “proconsumers” (Wilson, 2015), simultaneously functioning as a means to connect with a world outside, engage in conversations, and dive deeper into the television text. Of the target audience for Love Island, 16–24 years, 95% own a smart phone (Ofcom, 2018) enabling young people watch the show and simultaneously discuss it online, or simply see the show's highlights or discussions about it on social media. According to Jenkins (2016), this participatory culture means audiences are no longer passive consumers, but actively engage in, and collaborate across multiple platforms, sharing views with others. Young people are not passive watchers, and they discussed the processes behind reality TV, and the power of social and digital media to manipulate the behavior of both the participants and the audience. The older participants in particular were aware of this:

“*It keeps asking questions to keep engagement but they [the producers] don't care what they (the audience) think, it's just to get money, to boost figures and get sponsorship. Social media equals more interaction, more money” (FG age 21 F)*

Social media also plays a part in the selection of the contestants. Like the young people in Allen and Mendick's (2013) study, our respondents questioned ‘ordinariness' and authenticity of participants. It was obvious to our focus groups that those selected to be on Love Island were already “instafamous” Marwick (2015, 2016) prior to the show and their interest was to nurture their fame:

*They chose Insta models, people who are already famous who already have a particular life style. They don't choose average people. So if you think they are like you, you are fooling yourself [….]I think that the contestants weren't interested in love just wanted to become more famous (FG year 11 age 17 F)*.

This bought the “realness” of their relationship into question, and this idea of “fakeness” was discussed in more detail in terms of the relationships portrayed on Love Island (i.e., what is real on a reality TV show compared to what is real in lived experiences was questioned by the young people we worked with).

### Young People's Views of Relationships on Love Island: “True Love” or ‘a Toxic Battle”?

Whilst the majority of young people were aware of the lack of authenticity of the programme, some young people saw the show as a portrayal of positive relationships, where contestants can find “true love”:

One of the good things about it (love Island) is that they [the contestants] find true love well sometimes they get to meet new people and make new friends. (SG F year 8 age 13)

The positive aspects of the show tended to focus on friendships rather than romance (REF). However, they were aware that although some relationships were “genuine” the competitive nature of the programme complicates matters:

“*I think there is some genuine care but the game of the show makes things complicated and caused bad arguments. It shows how romance can blossom and you can see flirting and genuine connections and how relationship can develop” (FG age 17 F)*

The majority of the young people we spoke to were critical of the “fakeness” of the relationships shown and the fact that it is a game show with a financial incentive:

“I don't like because it's shit, it forces people to fall in love and get money and even if they don't fall in love [and the audience] find out, they still get money - the show is [meant to be] about trying to fall in love but it's really all about making money” (FG year 10 age 15 M)

Despite some positive comments, most young people saw the programme as promoting unhealthy relationships, with one young man commenting:

[it's] a toxic battle between the two sides instead of being normal and that's a bad thing to show kids and teenagers” (FG year 10 age 15 M)

As the show exaggerates the drama between contestants and their coupling, this creates the illusion that relationships are built on unrealistic conflicts between abuse and love. Bourne (2019) argues abusive relationships are portrayed as “gestures of love” with couples being rewarded for overcoming these to be “happily ever after”, which is not portraying a positive starting point for young people embarking on relationships. The young people in our focus groups however recognized that the relationships portrayed were not “equal” and were based on unhealthy behaviors:

It (Love Island) is bad; they change mates every week and swear, and argue at each other all the time. Someone is always crying because it feels like they're being forced into relationships and that's wrong. When you want to get with someone you both have to want it. It's like a joint decision. (SG year 8 age 13 F)

Some young people were also unhappy about the lack of diversity amongst contestants, not just in terms of body size and shape, but also because of the heteronormativity presented:

They only have boy girl relationships and that's not fair cos some of my mates are gay and it should really have gay contestants (SG year 9 age 13 M)

Young people wanted to see more diversity of relationships on the show to reflect their lived experiences. This awareness of the inauthenticity of the contestants suggest that the influence of programmes such as Love Island is not as great as media moral panics assume. Young people are active viewers who “negotiate the reality contract” of TV programmes (Allen and Mendick, 2013), being aware of the ways in which it constructs identities and invites the audience to make moral judgements around class, gender, and ethnicity. If the identities seem unauthentic, the viewers might begin doubting how “ordinary” or genuine the contestants truly are, which might result in young people relating less to the contestants and causing any influence to diminish. Young people were aware that the relationships may not be “real” and therefore they may not be influenced by what they see on TV:

It's like it's all forced for the cameras so it's not real it doesn't influence me because me and my mates know it's not genuine you know what I mean? (FG year 9 age 14 F)

It also brought into question young people's understandings of what relationships are, and the perceived “naturalness” of “falling in love.” For the young people, Love Island's relationships were not “real” because they were constructed on, and for, TV:

“*Don't like it because it's kind of like making relationships in a place where it's just like uncomfortable and not a place to make a solid relationship and you have to make decisions about relationships in front of other people and that's bad because it's unnatural like trying to grow food in a box. Relationships should just happen naturally without being forced (FG age 17 F)*

However, there was little discussion of what the “solid” relationship would be and how it would form. The idea that relationships happen “naturally,” is linked to late modernity concepts of romantic love (Giddens, 1992), without a recognition of social and structural constraints on relationships. This suggests that young people did not necessarily have the language and/or experience to frame their viewing within a wider context, or to question notions of “true love.” As one respondent commented, it presents a false view of relationships to younger viewers:

“*They trust many people who they don't really know and it isn't true love. In addition, Love Island is seen by hundreds of teenagers who take an incorrect view of what a relationship means” (FG age 16 F)*

As discussed earlier, adolescents and young adults are believed to be most susceptible to media messages depicting domestic abuse because they are still learning what behaviors are appropriate in romantic and sexual relationships (Arnett, 2000; Coyne et al., 2011). While some argue showing domestic abuse on television can serve as a means of educating viewers about the issue, it is also argued that the vast majority of portrayals on television make light of, or normalize, domestic abuse within intimate relationships (Kohlman et al., 2014).

Whilst a number of respondents thought Love Island presented a problematic view of relationships, many acknowledged it did enable young people to start conversations around “healthy” and “unhealthy” relationships, and this could be a positive move.

### Love Island as Relationship Education

The popularity of the show, and the associated social media attention, enabled young people to discuss relationships, and raised awareness of gaslighting and other early warning signs of abuse. Focus group participants stated they felt social media debates could empower young people to recognize and discuss abusive behavior:

when someone is trying to manipulate the other person or do gaslighting everyone recognizes it and sees it so everyone talks about it and brings awareness to unhealthy relationships then the media all joins in so the emotional trauma is talked about (FG age 17 F)

Our research showed that some young people are engaging with current debates about both the authenticity of Love Island and were able to recognize the examples of unhealthy relationships shown by the programme:

It's good it can teach young people about relationships what's good what's bad. It gets us taking about relationships in school and it's lets us see bad personalities (FG year 9 age 13 F)

Young people therefore were not passive consumers of reality TV, but interacted with social media to make decisions for themselves about relationships. However, whilst Love Island opened up a space for discussion, the focus was on “unhealthy” rather than positive relationships, young people discussed the “bad behavior,” more than the examples of healthy relationships, highlighting a need to provide more education on this. Based on their experience, some of the older focus group participants discussed how Love Island could be used in schools to teach about relationships:

Sex education for me was being told sex was all about rubbing and shaking a bit […]

Love island would be good in schools teaching the kids about relationships the conversations are a starting point, teachers could use the couples as examples when talking about things like what is a toxic relationship and what is gaslighting (FG, F 21)

Research shows that current RSE is not effective, is delivered too late, and is focussed on biology rather than relationships, consent, and sexual agency (Nocentini et al., 2010). As Peek and Beresin (2015, p. 178) state, “*Reality television is a stimulus for the ideals, values, behavior, and emotional development of children and adolescents”* and teachers need to be better informed about the impact of media exposure to young people. There is therefore potential for Love Island to be used to build both media literacy, and in sex and relationship education in schools. This has been raised by former contestants, commentators and sex education organizations (Bateman, 2018), for example former contestant Eyal Booker says that the programme is “*educational and can teach younger views about relationships, particularly teenage boys”* and some organizations, such as explain, have started running Love Island workshops in schools. In the concluding section, we discuss the roles schools play in providing PHSE, and if programmes such a Love island can contribute to relationship and sex education in school.

## Conclusion: Love Island and Relationship Education

Reality TV and its associated social media is just one of these ways in which models of intimate partner relationships are shown. The relationships shown on programmes such as Love Island, with its wide social media reach, are highly visible to young people, and present conversations about sex, relationships, and emotional abuse to a young audience. In our experience, young people's understanding of the relationships they see on reality TV are nuanced and complex; they understand that the relationships are “false” and constructed and edited for entertainment value but also recognize that some relationships were “bad” and unhealthy, and also engaged with and questioned behaviors on social media. Young people's understanding of what healthy and unhealthy relationships look like originate from many sources, including family, friends, peers, and the media. Notably absent from the discussions of sources of information for young people was the role of schools.

Discussions of Love Island are carried over from online discussions on social media from their bedrooms to face-to-face discussion in the school ground. Whilst Love Island does present some positive models of relationships, and in particular, of male friendships, it also portrayed elements of “toxic masculinity” and heteronormative gender roles and sexual relationships. Whilst young people recognize many behaviors are not “healthy,” and contestants' behavior is being questioned online by viewers themselves on social media, and by domestic abuse organizations, they may not have the skills and information to recognize the signs of unhealthy relationships, and speak out against abuse (Barter et al., 2009). The young people we worked with in schools often lacked an awareness of what romantic and sexual relationships entailed, and what the early warning signs of abuse are, and had limited reliable sources from which to gain information, often looking for information from social media and the internet.

Currently, relationship and sex education (RSE) is not compulsory across the UK, however, from September 2020, this will be a statutory requirement in all schools in England. Quality of provision varies widely, with Pound et al.'s (2016) systematic review finding more than a third of UK schools lacked good-quality RSE in 2013. In addition Ringrose (2016) argues RSE in England is “currently organized around sexual risk and danger in highly gendered and sexist ways,” which reflect, and play into, moral panics around the sexualization of girls and young women. However, despite its lack of status and variable quality, school-based RSE is seen as vital for safeguarding young people against domestic abuse and sexual exploitation.

Schools thus are environments where young people are socialized into gender norms and where significant amounts of gender-based harassment and IPVA goes unchallenged. Adolescence is “a crucial time when young women and men are developing their sexual identities' (Shaughnessy, 2007, p.1) and gender abuse emerges (STIR, 2015a). Policy and interventions in this area are underdeveloped and under-resourced (STIR, 2015b). However, evaluations of school-based domestic/sexual violence prevention interventions to date have suggested they enable children and young people to change their attitudes and perceptions of equality, respect, and consent and have a role in preventing relationship violence among young people (Wolfe et al., 2009; Barter et al., 2015). Likewise, evidence shows that media literacy interventions may also be an effective component in IPVA prevention efforts (Jeong et al., 2012), and violence prevention efforts have acknowledged the importance of being critical consumers of media.

Given that the media plays a crucial role in sexual socialization the inclusion of media literacy into relationship education programmes would help young viewers understand how the media, including reality TV, influences expectations about appropriate behavior (Rodenhizer et al., 2019). Bringing discussions of contemporary programmes such as Love Island into the classroom may be one way of engaging young people in discussions about healthy relationships and enabling them to be critical consumers of media.

In this context Love Island has the potential to be a starting point for discussions for relationship and sex education in schools using age and ability appropriate workshops with well-trained staff, which focus on identifying early warning signs in unhealthy relationships, can encourage young people to question dominant media portrayals, and challenge their current norm.

Twitter (2019) Data was collected from Twitter in compliance with the relevant terms and conditions.

## Data Availability Statement

The raw data supporting the conclusions of this article will be made available by the authors, without undue reservation, to any qualified researcher.

## Ethics Statement

Ethical approval was granted by the university; all participants were provided with written consent forms, as were their parents and guardians. We anonymized all the data, and refer only to young people's ages/school year and gender. Written informed consent to participate in this study was provided by the participants' legal guardian/next of kin.

## Author Contributions

All authors listed have made a substantial, direct and intellectual contribution to the work, and approved it for publication.

### Conflict of Interest

The authors declare that the research was conducted in the absence of any commercial or financial relationships that could be construed as a potential conflict of interest.

## Footnotes

^1^Love Island first aired in the UK in 2015 and has run for four season to date. Based in a villa in Spain contestants are invited to “couple up” and “re-couple” throughout the show with couples voted out by the public, and the couple with the most votes winning £50,000. In a final twist, one member of the winning couple can choose to either keep all the prize money, or share it with their partner.

^2^Disrespect Nobody (n.d.). https://www.disrespectnobody.co.uk/.

^3^*Big Brother* was a UK reality TV show on Channel 4. It aired between 2000 and 2018 attracting a wide spectrum of contestants knows as housemates who lived isolated from the outside world in a specifically designed house for a period of time. Housemates were evicted weekly by public voting. The last housemate in the house won a significant sum of money.

^4^Defined by APA (2018) to include: anti-femininity, achievement, eschewal of the appearance of weakness, and adventure, risk, and violence.

^5^A term used to describe traditional and stereotypical norms of masculinity, the expectations that boys and men are, amongst other things, aggressive, tough, and physically dominant (Flood, 2018) Gaslighting is one example of this.

^6^https://inews.co.uk/opinion/comment/love-island-adam-rosie-eyal-megan-relationship/ (accessed August 23, 2019).

^7^Maura's behavior toward Tommy when she forcibly kissed him after he said no, raised issues around consent, and led to the hashtag #doublestandard.

^8^The arts charity *Tender*. The Tender project uses art and drama to explore the early warning signs of abusive relationships.

^9^The Jeremy Kyle show was a UK tabloid talk/problem show, which ran between 2005 and 2019 on ITV. In total, it aired 3,320 times. It tackled difficult and emotional issues in a sensationalist manner. It was taken off air in June 2019 following the death of a participant a week after recording an episode of the show https://www.independent.co.uk/arts-entertainment/tv/jeremy-kyle-show-cancelled-stephen-dymond-death-guest-itv-latest-a8914471.html.
